# Profiling of Chemical and Structural Composition of Lignocellulosic Biomasses in Tetraploid Rice Straw

**DOI:** 10.3390/polym12020340

**Published:** 2020-02-05

**Authors:** Chen Chen, Zhixiong Chen, Jiajun Chen, Jiawei Huang, Huiling Li, Shaolong Sun, Xiangdong Liu, Aimin Wu, Bo Wang

**Affiliations:** 1 State Key Laboratory for Conservation and Utilization of Subtropical Agro-Bioresources, Guangzhou 510642, China; cc@cch3n.cn (C.C.); chenjiajun1001@126.com (J.C.); jiaweihuangkawy@gmail.com (J.H.); lihl@scau.edu.cn (H.L.); xdliu@scau.edu.cn (X.L.); wuaimin@scau.edu.cn (A.W.); 2 Guangdong Key Laboratory for Innovative Development and Utilization of Forest Plant Germplasm, College of Forestry and Landscape Architectures, South China Agricultural University, Guangzhou 510642, China; 3 The Key Laboratory of Plant Molecular Breeding of Guangdong Province, College of Agriculture, South China Agricultural University, Guangzhou 510642, China; 4 College of National Resources and Environment, South China Agricultural University, Guangzhou 510642, China; sunshaolong328@scau.edu.cn

**Keywords:** tetraploid rice, enzymatic saccharification, cell wall, lignocellulose, composition

## Abstract

The improvement of the saccharification of rice straw is one of the strategies to reduce the sophisticated pretreatment that results in high cost and is unfriendly to the environment. We explored the cell wall features in tetraploid rice and highlighted the enhanced saccharification of tetraploid with large biomass. Results showed that lignin content and S/G ratio reduced to 17.09% and 0.37, respectively, in tetraploid straw by the determination of the pyGC-MS method. After the pretreatment, the cellulose crystallinity index decreased from 63.22% to 57.65% in tetraploid straw, which is lower than that of pretreated diploid straw. Surface topological analysis of SEM images indicated that tetraploid straw was more susceptible to the pretreatment. Tetraploid straw showed a strong advantage in the process of enzymatic hydrolysis. The enzyme efficiency reached the highest value of 77.60%, and the rate of enzyme reaction was improved to make the reaction saturated earlier than conventional rice. We concluded that the high saccharification has resulted from the alteration of lignin and cellulose in tetraploid rice. Our research provides an improved green feedstock for bioenergy, and the tetraploid rice straw shows the potential utilization value in bioethanol production.

## 1. Introduction

As one of the renewable energy sources, biomass energy has advantages in a sustainable and favorable environment. Research of biomass energy mainly focused on lignocellulose, which consists of cellulose, hemicellulose, and lignin [1]. Rice is one of the world’s main crops, with its vast acreage and yield, but is also an excellent renewable energy resource. The biomass of straw cell wall is usually composed of 35%~50% cellulose, 20%~35% hemicellulose, and 10%~25% lignin in rice. Conventional rice is biologically diploid (2× = 24), and tetraploid rice (4× = 48) is produced by chromosome duplication. The polyploid plant is significantly different from diploid plants, including morphology, yield, total biomass, cell wall composition, cell size, etc. [2,3,4,5,6]. For biomass resources, the cell wall structure is often damaged by pretreatment to make it more susceptible to utilization, whereas, the polyploid plant has been characterized by changes in cell wall structure [4], revealing the potential roles in bioenergy fields.

Cellulose is the most abundant component in the cell wall. The cellulose molecule exhibits a linear distribution of stretching with β-d-pyranoglucoside and forms crystalline cellulose easily [7]. The low crystalline cellulose may affect the structure or accessibility of other polysaccharides, resulting in prompt degradation of cellulose [8]. Moreover, the cellulose H bonds functions to maintain the stability of cellulose. There are two kinds of H bonds in plant cellulose, which are intramolecular H bond and intermolecular H bond [9]. Breakage of these H bonds loosens cellulose structure, reduces the crystallinity and enhances the enzymatic hydrolysis efficiency of cellulose. Interestingly, it has been reported that the polyploid cellulose fiber length is higher than that of diploid poplar [4]. Studies in rice have shown that cellulose content is positively correlated with chromosome multiples, while lignin is negatively correlated [10].

Studies on lignin changes in polyploid plants have been reported in several pieces of literature. These changes mainly focused on the reduction in lignin content in the cell wall [4]. The structures of lignin in rice straw include syringyl lignin (S), guaiacyl lignin (G), and hydroxy-phenyl (H) lignin. Not only the ratio of syringyl lignin and guaiacyl lignin (S/G) affects the separation of lignin, but the lignin content also determines the sensitivity of the straw material to enzymes or other treatments [11,12,13]. Besides, the hemicellulose filled between cellulose and lignin can affect the cell wall utilization in plant as well [14]. The hemicellulose structure varies among different species or varieties [15]. Though the hemicellulose contents do affect the utilization of biological materials [10], the connection between hemicellulose and polyploids is unclear at present.

Compared to common diploid and transgenic plants, tetraploid rice has an enhanced yield, powerful biological potential, environmental adaptation, and considerable biomass [16]. Agronomic traits, such as larger grain size, stronger stem, and longer panicles of tetraploid, are genetically stable and identical. Whereas, the research on the chemical and structural composition of lignocellulosic biomasses in polyploid plants, especially the tetraploid rice, is missing. To explore the potential value of tetraploid rice in the bioenergy utilization, the cell wall compositions and enzymatic saccharification were characterized in tetraploid straw with the supportive evidence of XRD, FT-IR, SEM. The results demonstrated that the changes in cell wall structure resulted in the enhanced saccharification degree in tetraploid rice, making it the promising green biomaterials.

## 2. Materials and Methods

### 2.1. Plant Materials and Biomass Sample Preparation

**Figure 1:**
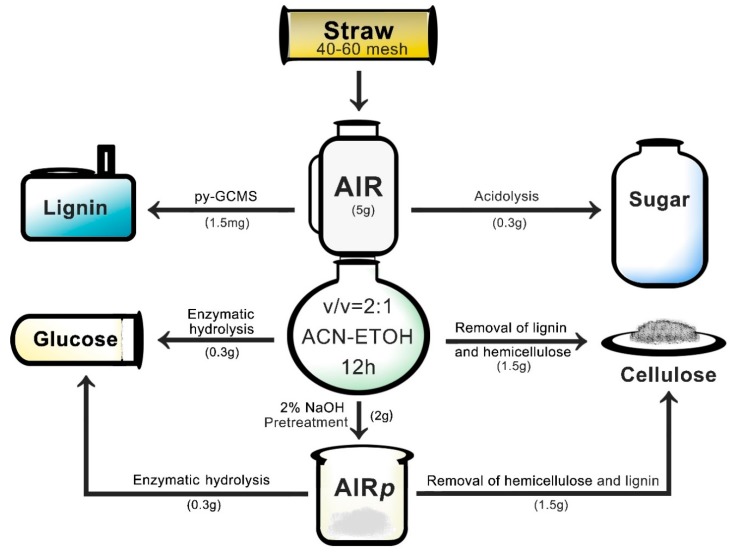
Schematic illustration of the experimental process.

Both the diploid rice and tetraploid rice were obtained from the experimental field of Agricultural College in South China Agricultural University (Guangzhou, China). The tetraploid rice (Aijiaonante-4×) was cultivated from Aijiaonante-2× by chromosome doubling using colchicine treatment. Ten individual plants were harvested at mature stages. Leaves, including blades and sheathes, were removed from stems. The stems were dried at 60 °C for 24 h, then the dried straw segments were crushed by a crusher for 1 min and screened with sieved nets (40 to 60 mesh) for further analysis. The samples were dewaxed by the soxhlet extraction method for 5 h with 2:1 (*v*/*v*) acetone-ethanol at 90 °C, then air-dried at room temperature to obtain the alcohol-insoluble residue (AIR). This process is shown in Figure 1.

### 2.2. Cell Characteristic Observation by Microscope

The straw slices (65 μm) were obtained by vibratome (Leica VT1000 S, Leica Microsystems, Buffalo Grove, IL, United States) and dyed with 0.2% toluidine blue (m/v). After 1 min of dyeing, the excess dye was washed with 70% ethanol. The dyed slices were observed under a scanning microscope (Wanbang M8). Cell number and cell size were calculated by using ImageJ software (LOCI, University of Wisconsin, WI, USA, https://imagej.nih.gov/ij/).

### 2.3. Material Composition Analysis

Cell wall composition analysis was performed using a stepwise acid hydrolysis method. AIR (0.3 g) was taken and acidified by using 72% sulfuric acid (3 mL) at 30 °C for 1 h and then treated at 121 °C for 1 h with the addition of 84 mL of deionized water. After cooling, it was filtered using a sand core funnel (G4). The diluted filtrate and the fine residue were filtered through 0.22 μm nylon membrane for monosaccharide composition analysis by ion chromatography. The ion chromatogram (Metrohm 940, Herisau, Switzerland) equipped with the CarboPac PA10 column (2 mm × 250 mm, Dionex, Sunnyvale, CA, USA) and coupled with a PAD detector was used for quantification. NaOH (20 mM) was added as an isocratic eluent and eluted at a flow rate of 0.5 mL/min for 20 min. The program was set to 0–75 mM NaAc 15 min for gradient elution, 200 mM NaOH 10 min for washing, and then 20 mM NaOH for re-equilibration. A calibration curve was established to calculate the quantification of the monosaccharide.

The acidified elute was diluted ten times, and the percentage of the sample content was calculated by the following formula:

(1) $$\%Comtent=\frac{D\times V_{1}\times 10^{-6}\times P\times 0.9}{M}\times 100\%$$

where *D* was the dilution factor of acidified elute (10×), *V*_1_ was the total liquid volume used for hydrolysis (*V* = 86.663 mL), *P* was the concentration determined by ion chromatography (mg/L), and the *M* was the weight of AIR.

### 2.4. Isolation of Hemicellulose and Cellulose

Lignin was removed from AIR samples using 0.1 mol/L sodium chlorite-acetic acid solution (acetic acid, 1:100(*v*/*v*)) for 4 h at 75 °C for hemicellulose and cellulose isolation. The hemicellulose was extracted at 50 °C using 0.5 M KOH. The solid-liquid mixture was filtered and separated into pellet and filtrate. The filtrate was neutralized with acetic acid, and three volumes of 95% ethanol were added to precipitate hemicellulose. Finally, the liquid was further lyophilized to collect hemicellulose. On the other side, the pellet was used for cellulose extraction. The cellulose was obtained by KOH gradient washing and eluted with 80% DMSO at 80 °C for 5 h to remove the remaining hemicellulose.

### 2.5. The Ash Determination

The determination of ash was carried out by a rapid ashing method using a muffle furnace. The ash dish was burned to constant weight before the determination. AIR (1 g) was evenly spread in the container, and the ash dish was slowly pushed into a muffle furnace at 850 °C for 40 min. The weight was recorded after cooling to room temperature.

### 2.6. Enzymatic Hydrolysis

The hydrolyzed samples consisted of AIR and pretreated AIR by 2% NaOH (60 °C, 6 h). AIR (0.3 g) was dissolved in 30 mL 0.1 M NaAC-HAC buffer (pH = 4.5). The cellulase (10 FPU/g) was chosen for the enzymatic hydrolysis analysis at 55 °C for 4, 12, 24, 48, and 72 h, respectively. After the enzymatic hydrolysis, the reaction liquid was deactivated and filtered through a 0.22 μm filter. The concentrations of glucose were determined by ion chromatography. The hydrolysis efficiency of the enzyme was calculated as follows:

(2) $$\%Enzymatic\;digestibility=\frac{\left(D\times R\times V_{2}\right)}{\left(P\times V_{1}\right)}\times 100\%$$

where *R* was the released glucose concentration by ion chromatography. *V*_2_ was a volume of the enzymatic reaction liquid. *V*_1_, *D*, and *P* have the same meanings as above (see Section 2.3).

### 2.7. Scanning Electron Microscope (SEM) Observation

The sample was coated with a thin gold film. The model of the scanning electron microscope used was Zeiss HD (Zeiss Sigma HD, Jena, Germany) at 5 kV acceleration voltage. The surface morphology of the straw was observed. Then, the energy spectrum sweeping (point scan) was performed to obtain semi-quantitative data of Si, Al, and Cd elements.

### 2.8. Crystallinity Measurement

The crystallinity of the straw was analyzed by X-ray diffraction (XRD) (D8 Bruker, Madison, WI, USA) at 40 kV and 30 mA with copper radiation. The scanning angle was in the range of 5° to 50°, and the scanning speed set to 2°/min. The crystallinity index (*CrI*) was calculated according to the following formula:

(3) $$CrI\left(\%\right)=\left(I_{002}-\frac{I_{Am}}{I_{002}}\right)\times 100\%$$

where *I*_002_ was the highest value of the peak at 22.04 (2θ). *I_Am_* is the maximum value of the amorphous cellulose peak at 18.41 (2θ).

### 2.9. Fourier Transform Infrared Spectroscopy (FT-IR)

The sample was ground with KBr in a ratio of 1:100 (*m*/*m*) to a fine powder and dried at 50 °C for 12 h. The mixed sample was pressed into a sheet in compressing equipment for further FT-IR analysis (VERTEX 70, Ettlingen, Germany). A total of 40 scans were performed at a resolution of 4 cm^−1^. The absorption spectra were obtained from 2000 to 500 cm^−1^.

### 2.10. The Measure of Lignin Content and Composition by Pyrolysis GC/MS

AIR (1.5 mg) was pyrolyzed in an EGA/PY-3030D pyrolyzer (Frontier Laboratories, Saikon Koriyama, Japan) connected to a QP2010 GC/MS system (Shimadzu, Kyoto, Japan) with a DB-5 capillary column (30 mm × 0.25 mm × 0.25 μm, Agilent, Santa Clara, CA, USA). The pyrolysis was heated from 200 °C (maintained for 1 min) to 600 °C (maintained for 10 s), and the rate of temperature rise was 20 °C/min. The GC was heated from 40 to 280 °C, the rate of temperature rise was 5 °C/min, and maintained 10 min at 280 °C. The flow rate of helium was 2 mL/min. The results were identified for compensation according to the standards of the Wiley and National Institute of Standards and Technology (NIST) libraries [17]. Finally, the peak area was calculated and normalized.

The composition results of straw by pyGC-MS include carbohydrate related (C), guaiacyl units (G), syringyl units (S), and p-hydroxyphenol units (H). The generic benzene derivatives, which without OH group on the aromatic ring (but most probably originated from lignin related compounds) (P), a substance that knows spectra information (unknown identification) (U) and unknown spectra (cannot be lignin or carbohydrates) (0), also were determined. Each substance was expressed as a percentage of the total (C+G+S+H+P+U+0). The total lignin refers to G+S+H+P.

## 3. Results and Discussion

### 3.1. Phenotypic Features of the Tetraploid Straw Cell Wall

**Figure 2:**
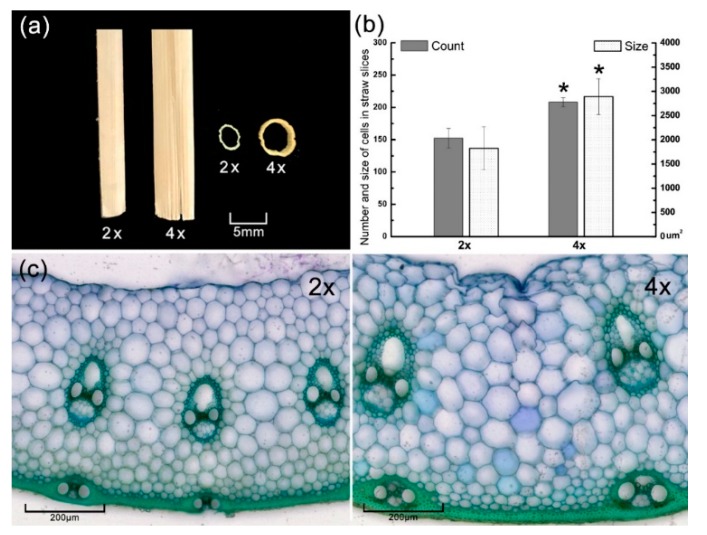
Straw morphology in diploid (2×) and tetraploid (4×) rice. (**a**) Straw phenotype. (**b**) Cell number and cell size calculated by ImageJ. (**c**) Straw slice dyeing with toluidine blue.

Tetraploid rice and diploid rice have significantly different agronomic traits, such as an increase in biomass and yield [18,19]. In order to explore the phenotypic feature of the cell wall in tetraploid rice straw, the slices from the fresh straw were observed using the microscope. The number and size of cells were analyzed by ImageJ software. Results showed that tetraploid rice had more cells and larger xylem vessels, which correlates to the large cell-size of tetraploid (Figure 2a,b). The most utilized ingredients of straw cell walls were epidermis, phloem, and xylem, which were thicker and more numerous (Figure 2c). It implies that the tetraploid straw has more available ingredients than conventional rice.

### 3.2. Composition Analysis of the Tetraploid Straw

**Figure 3:**
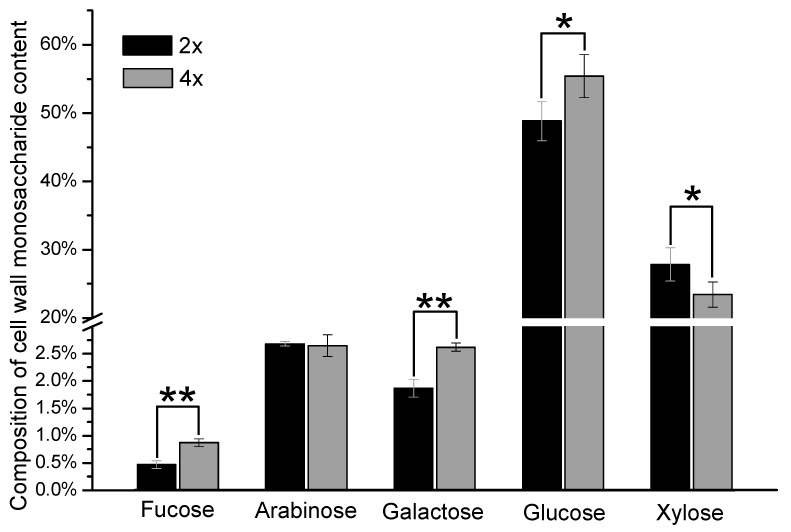
Monosaccharide component analysis of cell wall of tetraploid rice (4×) and diploid rice (2×). The significance analysis followed the student’s *t*-test, ** indicates *P* ≤ 0.01, * indicates *P* ≤ 0.05.

It would be interesting to know whether these phenotypic changes alter the cell wall structure in the tetraploid. Therefore, polysaccharide components, lignin contents, and ash were investigated. The results of polysaccharide components presented the content of monosaccharides. Around half of the substance in the cell wall was cellulose, which contributed by the highest level of glucose. Glucose content in the tetraploid was 55.42%, which is higher than that in the diploid (Figure 3). Xylose is the major sugar from hemicellulose, and it showed a significant decrease in tetraploid (Figure 3). The level of hemicellulose was 4.44% less than the diploid (Table 1). Arabinose plays the role of modification xylan main chain of hemicellulose polysaccharides. No significant change in arabinose content was detected, which means that the ratio of xylose and arabinose decreased. These results indicate a low branching degree of hemicellulose in the tetraploid.

The lignin was measured by pyGC-MS method. The results showed that the lignin content and S/G were significantly lower than the diploid (Table 1). In previous studies, the changes in cell wall composition of tetraploid plants, including a decline in lignin content [4], were found to be consistent with our results.

As the ash in other polyploid plants negatively correlated with cellulose [10], the ash content of tetraploid rice was detected. The content of tetraploid rice straw ash was only 10.15% (Table 1), while the cellulose content increased (Figure 3). This data is consistent with the previous finding [10]. In summary, the tetraploid straw showed particular cell wall composition with high cellulose and low lignin and ash, implying that tetraploid rice might be an improved feedstock of bioresources.

### 3.3. Optimum Enzymatic Hydrolysis Ability of Tetraploid Straw

**Figure 4:**
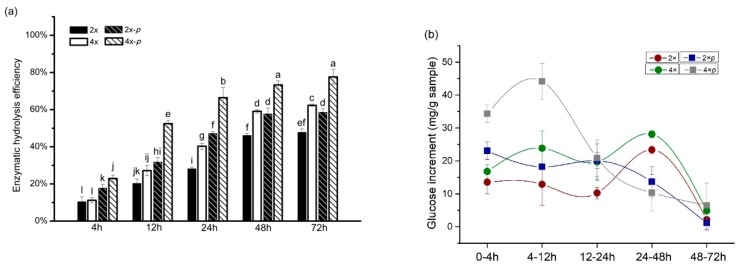
Saccharification of tetraploid rice straw. (**a**) The efficiency of enzymatic hydrolysate of tetraploid (4×), diploid (2×), tetraploid after pretreatment (4×-*p*), and the diploid after pretreatment (2×-*p*). (**b**) Glucose increment over the reaction periods. It reflects the rate of glucose products during enzymatic hydrolysis. The significance analysis followed LSD-test.

Enzymatic hydrolysis of tetraploid was assessed with the AIR and the 2% NaOH pretreated AIR. The enzymatic efficiency and the glucose increment were two parameters to present the enzymatic hydrolysis performance. At the initial stage of the enzymatic hydrolysis reaction, the enzymatic efficiencies of tetraploid and diploid were only 11.22% and 10.45%, respectively (Figure 4a), and they raised to 22.94% and 17.72% after alkaline treatment, respectively. These enzymatic reactions gradually saturated within 72 h. The enzyme efficiency of tetraploid at the final stage of hydrolysis reached 62.31%, which showed a 14.51% increase compared to the diploid. Furthermore, the final efficiency of pretreated tetraploid was as high as 77.60%, an increase of 19.80% compared to pretreated diploid (Figure 4a). Our results indicated that tetraploid straw shows significant advantages of enzymatic hydrolysis both before and after pretreatment. Furthermore, the hydrolysis efficiency caused by the pretreatment was 15.29% in tetraploid straw, which was a 4.55% higher than the diploid, indicating that the advantages of tetraploid in hydrolysis could be enhanced by pretreatment. These advantages could be due to structural differences in tetraploid cell walls.

In addition to the hydrolysis efficiency, glucose increment was statistically analyzed with the released glucose per gram of samples during hydrolysis. The period at 4–12 h showed fastest increase of pretreated tetraploid straw. The amount of tetraploid glucose increment was much higher than diploid rice straw over this previous period. This implies that tetraploid rice straw can interact with enzymes more quickly under the same enzyme contents and reaction conditions. Not only that but also non-pretreated tetraploid in the 4–12 h period had a higher increment than pretreated diploid (Figure 4b), which further confirmed the tetraploid straw showed dominant advantage on the hydrolysis. In addition, an increased peak was only found at 24–48 h in the non-pretreated rice straws (Figure 4b), which might be due to low velocity without the pretreatment. In brief, pretreated tetraploid showed a higher reaction ratio, especially in the earlier stages of the hydrolysis, which increased significantly more glucose than other samples (Figure 4b). The high reaction ratio in tetraploid rice straw could be due to structural differences of tetraploid cell walls as well.

In general, changes in cell wall structure include content and structure changes of lignin, cellulose, and hemicellulose. The tetraploid showed decreased lignin and ash content, changes of lignin structure, and increased cellulose content (Figure 3, Table 1). As previously published, these changes can significantly affect the enzymatic hydrolysis process [20,21,22,23,24], which is consistent with our results in the tetraploid straw.

Recent studies have explored the positive effect of Cd on saccharification efficiency [25], which may be related to the structure of the cell wall. Cd elements were semi-quantitatively determined, coupling with Si and Al as the reference elements (Table 2). Surprisingly, the relative proportion of Cd in tetraploid rice straw cellulose far exceeds that of diploid straw. This also supports the advantages of tetraploid in the enzymatic process.

### 3.4. Observation of Surface Topography before and after Tetraploid Pretreatment

**Figure 5:**
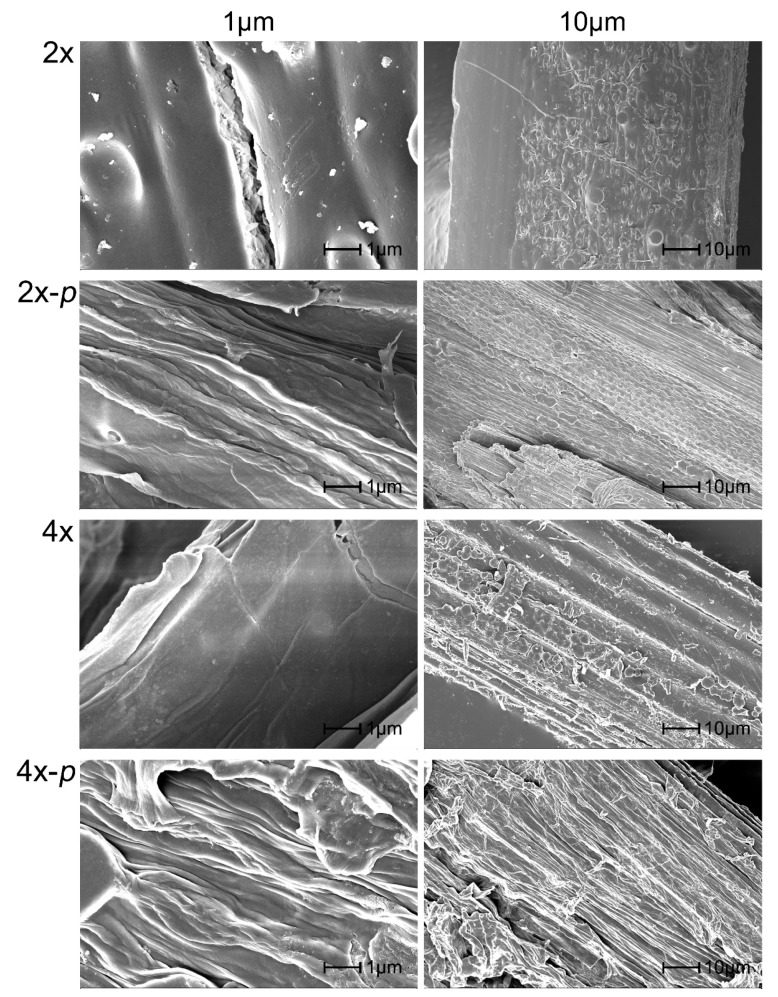
Scanning electron microscope of tetraploid (4×), diploid (2×), tetraploid after pretreatment (4×-*p*), and the diploid after pretreatment (2×-*p*).

Because changes of lignin in the tetraploid cell wall might inevitably lead to differences in surface topography, the straw surface and the pretreated sample were observed by SEM. The surface of the diploid straw was smooth, while the tetraploid straw had a rich texture. After pretreatment, the surface of diploid and tetraploid straw showed noticeable swelling and deep folds caused by the removal of some components, such as lignin. The degree of swelling and pleats of the tetraploid was higher than that of diploid, resulting in an increase in contact area with the enzyme (Figure 5). The presence of these ordered textures allows the tetraploid to have not only better physical-mechanical properties in plants but also increases the contact area with the enzyme during the hydrolysis reaction. The surface topography of the tetraploid rice might result from the low lignin content and S/G in tetraploid, shown in Table 1. Therefore, lignin changes might correlate with the loosen cell wall, which makes the tetraploid straws are much more susceptible to the pretreatment process.

Furthermore, the cellulose obtained by removing most of the lignin and hemicellulose were scanned using SEM. This result confirmed that the surface roughness of cellulose was similar and swelling occurred in both tetraploid and diploid after the pretreatment (Figure S1).

### 3.5. Fourier Transform Infrared Analysis of the Tetraploid Straw

**Figure 6:**
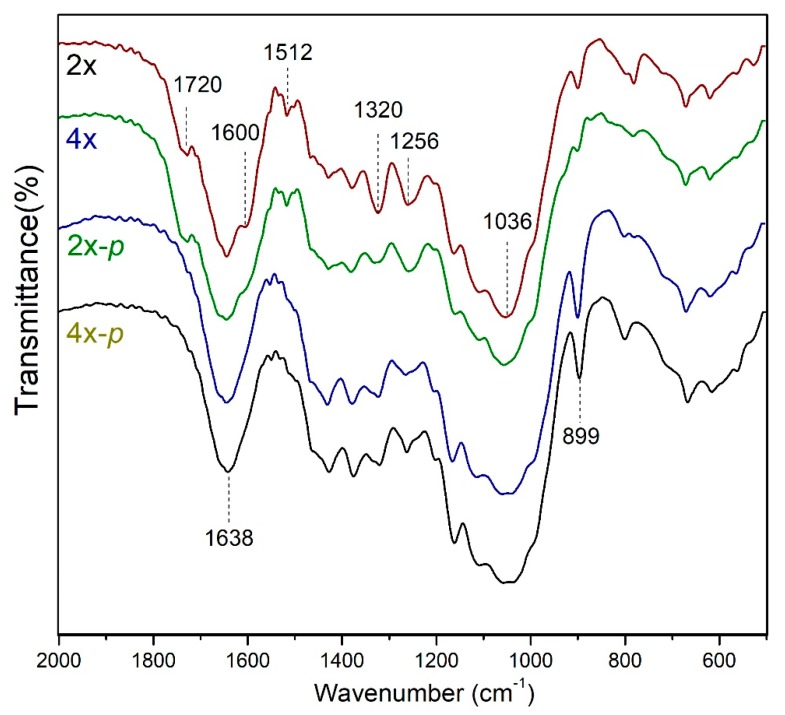
Fourier transform infrared analysis of tetraploid (4×), diploid (2×), tetraploid after pretreatment (4×-*p*), and the diploid after pretreatment (2×-*p*).

The straws before and after pretreatment were spectrally detected by FT-IR to verify the different bands. Three lignin-related absorption bands, 1512, 1320, and 1256 cm^−1^ were detected (Figure 6). Compared to the untreated diploid straws, the untreated tetraploid sample showed a slight decrease at 1512 cm^−1^ due to aryl-H vibrations of lignin [26]. The absorption peaks at 1320 cm^−1^ and 1256 cm^−1^, respectively, originated from syringyl units and guaiacyl units, went down as well. The intensity of these absorption peaks indicates that the tetraploid lignin level was below the diploid. Furthermore, the FT-IR spectrum showed less S/G in tetraploid than that in diploid rice (Figure 6). This result further confirmed that the S/G is low in the tetraploid compared to the diploid by pyGC-MS analysis (Table 1). The absorption peak at 1512 cm^−1^ disappeared after pretreatment, indicating the lignin was dissolved during the pretreatment.

The absorption bands for polysaccharides were found in the FT-IR spectrum as well. The absorption of diploid rice at 1600 cm^−1^ included not only aromatic skeleton vibrations but also polysaccharide-derived vibrations, and this absorption peak gradually disappeared on the tetraploid and pretreated samples. In addition, the large absorption peaks at 1638 and 1036 cm^−1^ were carbonyl stretching and polysaccharide vibrations, respectively [27,28], which were present in all samples. The polysaccharide bands showed no significant changes in FT-IR results.

### 3.6. Analysis of Cellulose Structure in Tetraploid Straw

**Figure 7:**
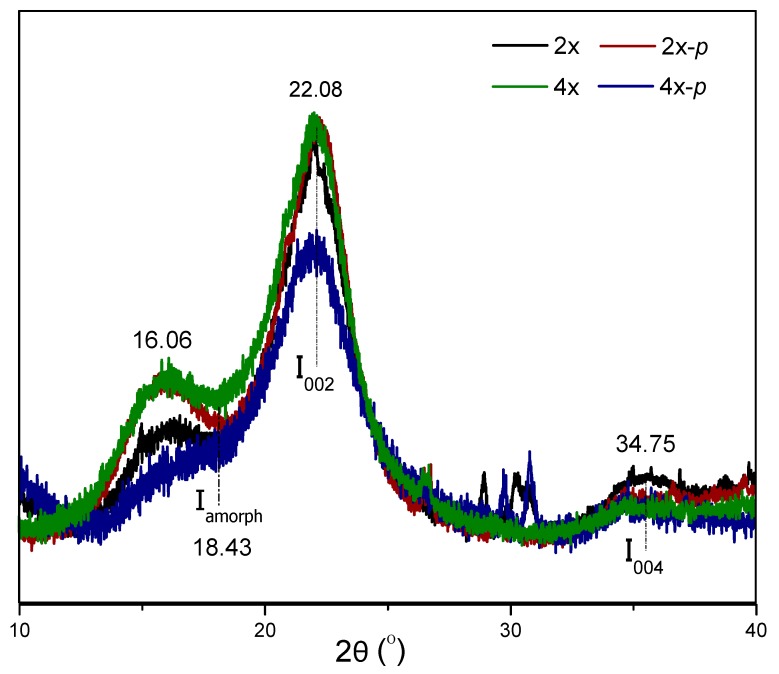
Diffraction of X-rays of tetraploid (4×), diploid (2×), tetraploid after pretreatment (4×-p), and the diploid after pretreatment (2×-p).

To further investigate cellulose crystallinity of the tetraploid rice, the powder of AIR was measured by XRD. Three peaks at 2θ = 16.06, 22.08, and 34.75 were characteristic peaks in the crystalline region of cellulose I (Figure 7). Combined with the valley of the amorphous region at 2θ = 18.43, the crystallinity index was calculated. The crystallinity index of the tetraploid was 63.22%, and it was 8.07% lower than that of the diploid. After the pretreatment, the crystallinity indexes decreased to 67.37% and 57.65% in diploid and tetraploid straws, respectively. In particular, the pretreated-tetraploid crystallinity was 9.72% lower than that of the pretreated-diploid. The low crystallinity makes cellulase accessible to cellulose hydrolysate and shows high saccharification efficiency (Figure 4a).

Besides cellulose crystallinity, the functional groups in cellulose also contributed to enzymatic saccharification. To explore the functional group changes of the cellulose in the tetraploid, the fingerprint region of the FT-IR spectra of the purified cellulose from rice straw was determined. Two bands of 897 and 1381 cm^−1^ were observed, which exhibit a typical -CH stretching in cellulose I and asymmetric deformation of -CH from polysaccharide, respectively [29,30,31]. Moreover, the peak at 1642 cm^−1^ corresponds to the stretching vibration of cellulose-bound water, the peaks of 1162 and 1110 cm^−1^ have been classified into asymmetric stretching of C-O-C and C-O elongation, respectively [32]. All these cellulose bands showed no structural difference between tetraploid and diploid straw. They share the same characteristic peak shape and slightly different peak intensity (Figure S2), indicating that the functional group of cellulose is not the dominant factor for the higher saccharification efficiency of tetraploid. Furthermore, the characteristic absorption peak of the C=C aromatic ring representing lignin appeared at 1428 cm^−1^ [30,33], indicating that there is still a small amount of lignin residue in the process of de-lignification.

## 4. Conclusions

Rice straw is an essential green material for biomass utilization, while its utilization of conventional rice straw requires complex pretreatment processes and high cost during the pretreatment. This study revealed that tetraploid rice straw showed enhanced saccharification, implying the potential to simplify the pretreatment and reduce the cost. The innate reduction in lignin content and changes in syringyl units and guaiacyl units directly make the straw more accessible to be pretreated, thereby reducing the use of pretreatment. Besides, decreased cellulose crystallinity can further improve the efficiency of utilization.

## Figures and Tables

**Table 1:** Composition of tetraploid straw cell wall.

Sample	Hemicellulose (%)	Lignin (%)	S (%)	G (%)	S/G	Ash (%)
2×	30.51 ± 2.15	19.41 ± 0.42	3.32 ± 0.09	8.59 ± 0.17	0.40 ± 0.007	14.32
4×	26.07 ± 1.76 *	17.09 ± 0.51 ***	2.83 ± 0.11 ***	7.51 ± 0.24 **	0.37 ± 0.009 **	10.15 **

The significance analysis followed student’s *t*-test, *** means *P* ≤ 0.001, ** means *P* ≤ 0.01, * means *P* ≤ 0.05. The S (%) and G (%) data means the proportion of Syringyl units or Guaiacyl units in total composition (C+G+S+H+P+U+0). 4× refers to tetraploid rice, and 2× refers to diploid rice.

**Table 2:** Elemental normalization semi-quantitative analysis.

Sample	Chemical Element	Standard Sample	Uniformization (Weight)%	Uniformization (Atomicity)%
2×	Al	Al2O3	ND ^a^	ND ^a^
	Si	SiO2	99.55	99.89
	Cd	Cd	0.45	0.11
4×	Al	Al2O3	0.05	0.05
	Si	SiO2	94.8	98.61
	Cd	Cd	5.15	1.34

^a^ ND, not detected.

## Supplementary Materials

The following are available online at https://www.mdpi.com/2073-4360/12/2/340/s1, Figure S1: Surface morphology of cellulose purified from tetraploid (4×), diploid (2×), tetraploid after pretreatment (4×-*p*), and the diploid after pretreatment (2×-*p*) by scanning electron microscope. Figure S2: FT-IR spectrum of cellulose of tetraploid (4×), diploid (2×), tetraploid after pretreatment (4×-*p*), and the diploid after pretreatment (2×-*p*).
