# Supplementary material for: Profiling of Chemical and Structural Composition of Lignocellulosic Biomasses in Tetraploid Rice Straw
Source: Polymers (Basel). 2020 Feb 5;12(2):340. doi: 10.3390/polym12020340 (PMC7077374; doi:10.3390/polym12020340)
Supplement: Supplementary file 1 [file polymers-12-00340-s001.pdf]

Profiling of Chemical and Structural Composition of Lignocellulosic Biomasses in Tetraploid Rice Straw

Chen Chen [1,2,†], Zhixiong Chen [3,†], Jiajun Chen [1,2], Jiawei Huang [1,2], Huiling Li [1,2], Shaolong Sun [4], Xiangdong Liu [1,3], Aimin Wu [1,2] and Bo Wang [1,3,*]

[1] State Key Laboratory for Conservation and Utilization of Subtropical Agro-bioresources, Guangzhou, 510642, China; cc@cch3n.cn (C.C.); chenjiajun1001@126.com (J.C.); jiaweihuangkawy@gmail.com (J.H.); lihl@scau.edu.cn (H.L.); xdliu@scau.edu.cn (X.L.); wuaimin@scau.edu.cn (A.W.)

[2] Guangdong Key Laboratory for Innovative Development and Utilization of Forest Plant Germplasm, College of Forestry and Landscape Architectures, South China Agricultural University, Guangzhou, 510642, China

[3] The Key Laboratory of Plant Molecular Breeding of Guangdong Province, College of Agriculture, South China Agricultural University, Guangzhou 510642, China; chenzx@scau.edu.cn

[4] College of National Resources and Environment, South China Agricultural University, Guangzhou 510642, China; sunshaolong328@scau.edu.cn

* Correspondence: bowang@scau.edu.cn

† These authors contributed equally to this work.

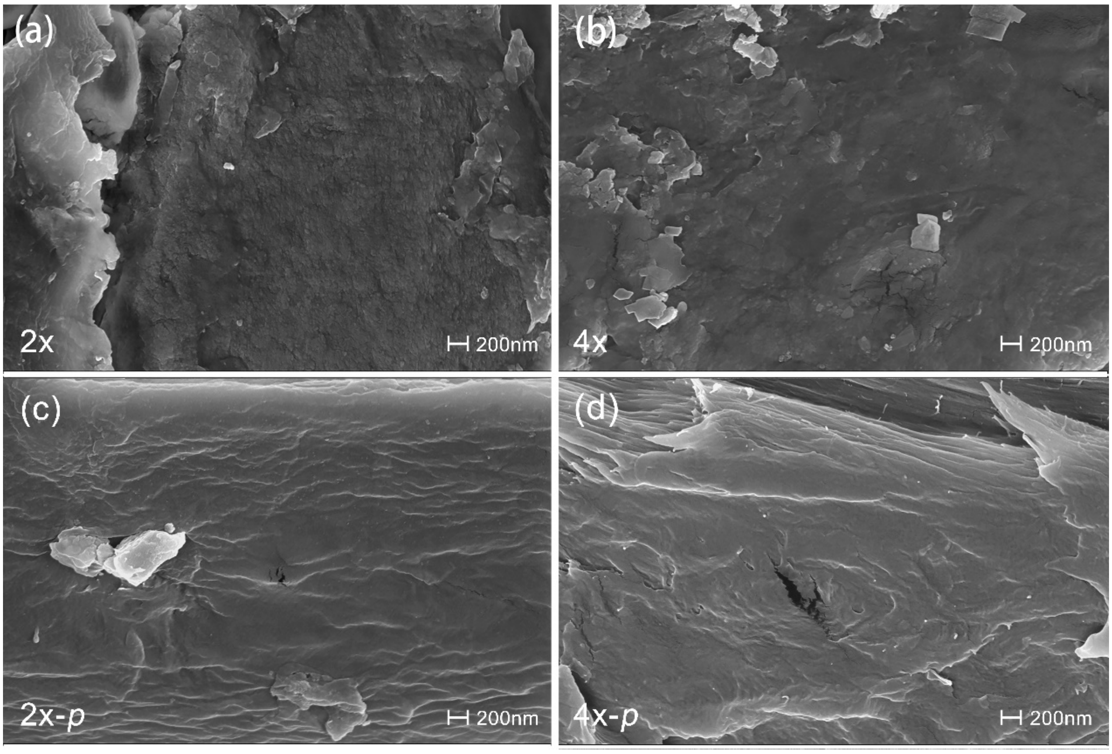

Figure S1. Surface morphology of cellulose purified from tetraploid (4x), diploid (2x), tetraploid after pretreatment (4x-p) and the diploid after pretreatment (2x-p) by scanning electron microscope.

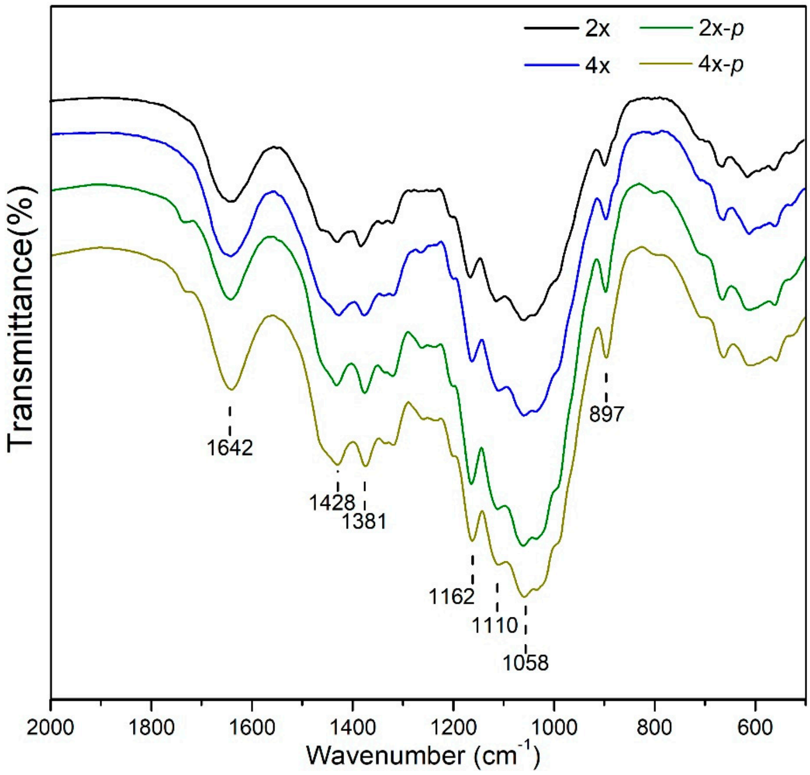

Figure S2. FT-IR spectrum of cellulose of tetraploid (4x), diploid (2x), tetraploid after pretreatment (4x-*p*) and the diploid after pretreatment (2x-*p*).
